# Sir Henry Halford

**Published:** 1844-09

**Authors:** 


*Sir Henry Halford.*—Among the notables of the profession who have lately paid the debt of nature, is Sir Henry Halford, whose career lasted half a century. His name originally was Vaughan, and as such was advantageously known in the medical world (of London). It was changed to Halford in consideration of a fortune. He was knighted by George IV., and would have been made a peer, could a precedent have been found for such an act. Lawyers are ennobled by the scores for all kinds of services; a man with a freehold in "fat acres," or who can command the representation of two or three boroughs, may also ingratiate himself with the minister of the day, and be made a peer; but for a physician or a surgeon, who may save the life of his sovereign by his skill, or the lives of thousands of his fellow subjects by his well-directed and timely applied knowledge, to become more than a mere baronet, would be preposterous. It is not so written in the book of precedents. A successful soldier of fortune may rise from an ensign to a duke, and take his seat in the House of Lords; but a well-educated and benevolent physician, whose endeavor it has been to save lives, to enlist the harmonizing influences in favor of his fellow-men, and by his skill and address to revive the drooping spirits of an entire army or a garrison, cannot hope for such honors.

Sir Harry Halford was a popular and fashionable physician, a man of moderate capacity and of respectable literary attainments. His manners and address gave him vogue, and tact was the word by which his friends and admirers expressed their opinion of his skill, when they dared not speak of his talents.

Dr. Paris has succeeded Sir Henry in the post of President of the College of Physicians.—*Ibid.*

